# Supplementary figures and images for: Assessment of the Performances of the Protein Modeling Techniques Participating in CASP15 Using a Structure-Based Functional Site Prediction Approach: ResiRole
Source: Bioengineering (Basel). 2023 Nov 30;10(12):1377. doi: 10.3390/bioengineering10121377 (PMC10740689; doi:10.3390/bioengineering10121377)

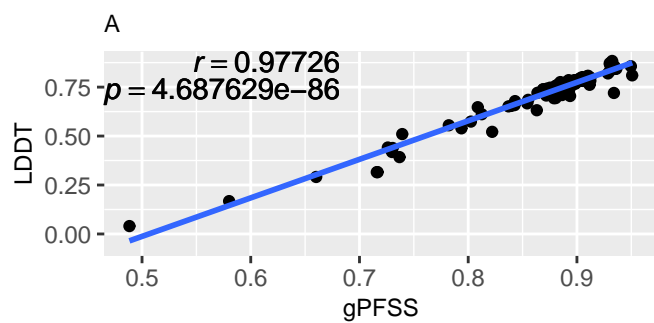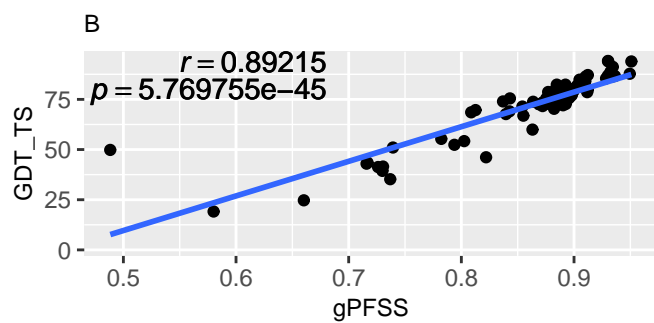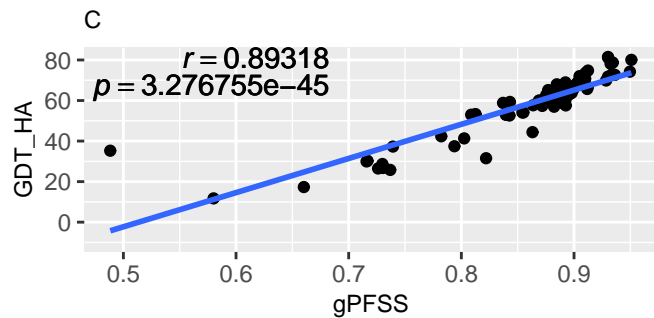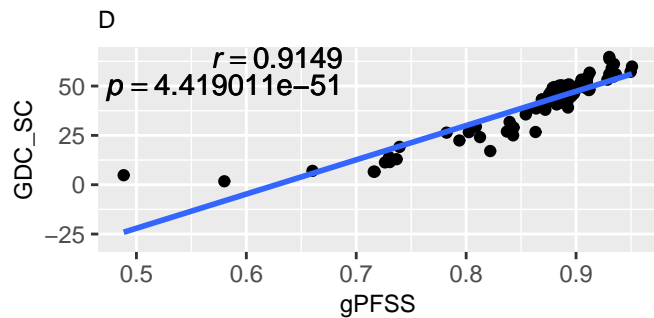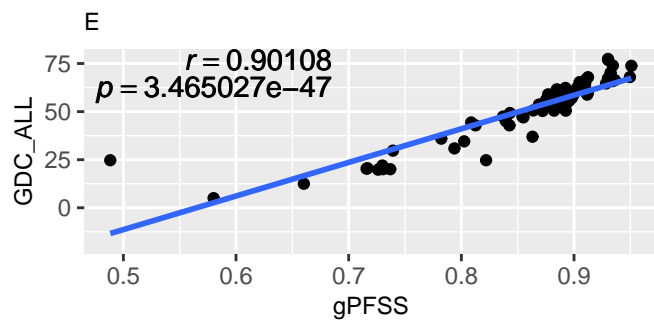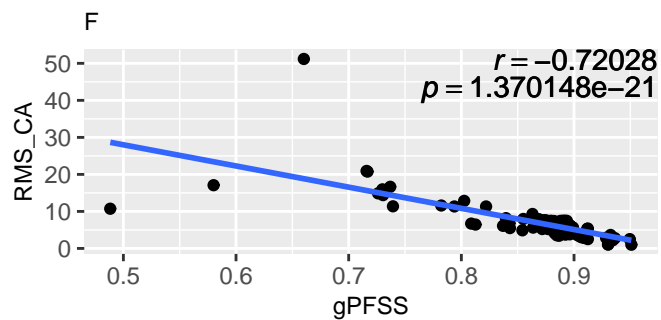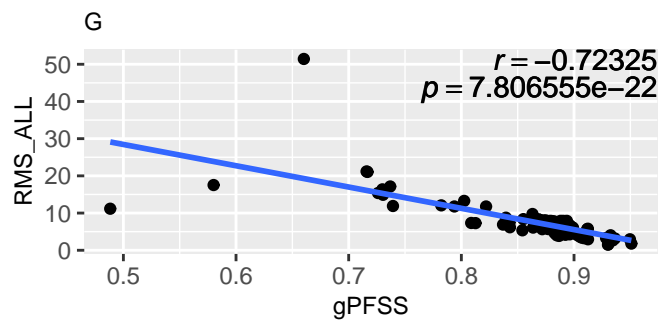

Supplement: Supplementary file 1 [file bioengineering-10-01377-s001.zip › Figure_S1_Plot_of_Metrics_gPFSS_All.pdf]

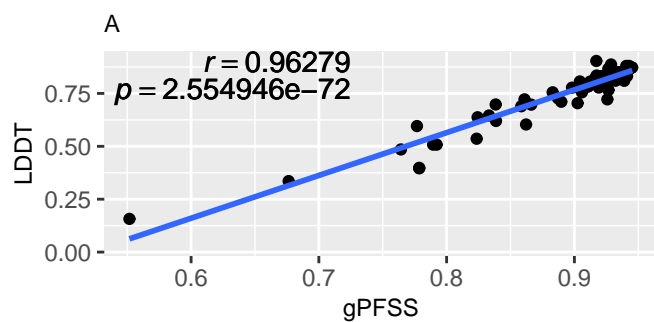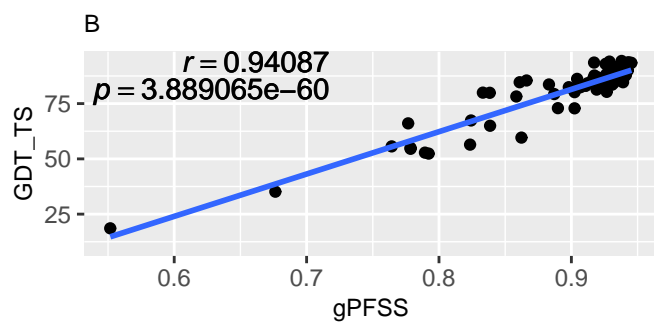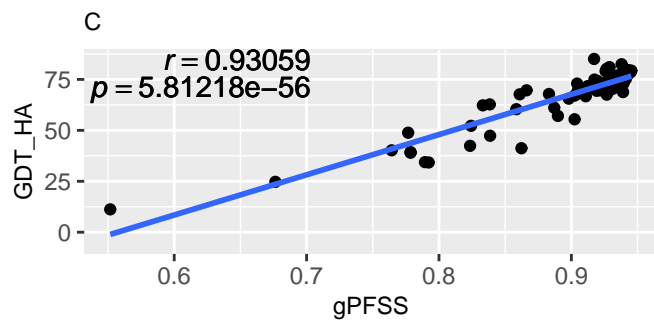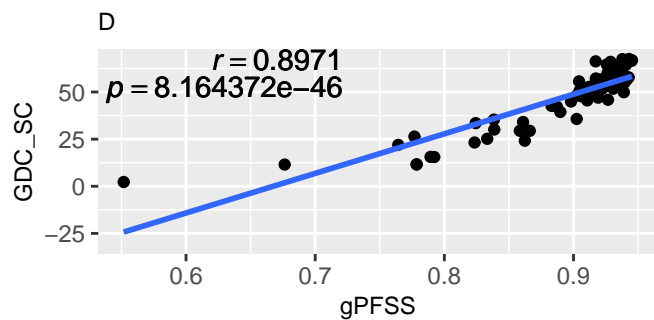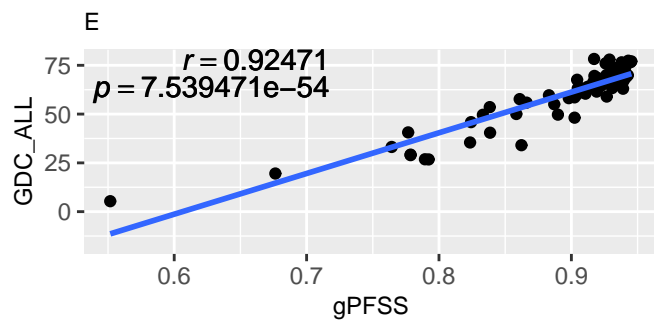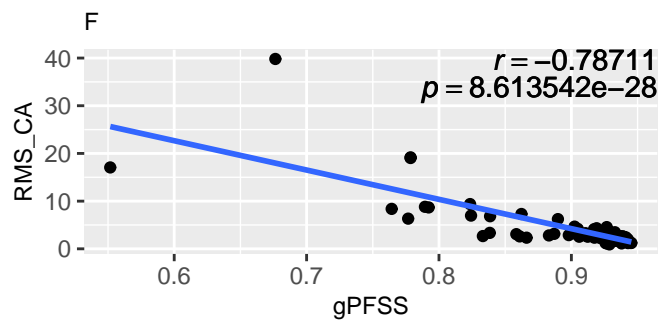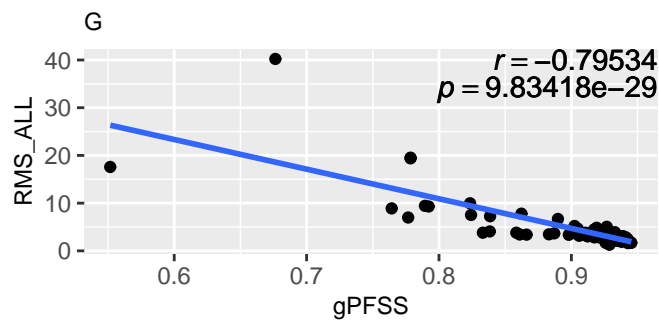

Supplement: Supplementary file 1 [file bioengineering-10-01377-s001.zip › Figure_S2_Plot_of_Metrics_gPFSS_TBME.pdf]

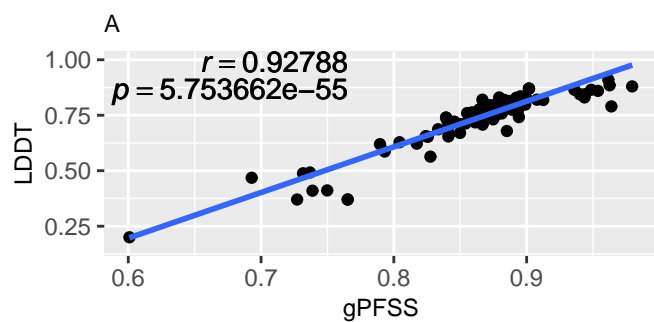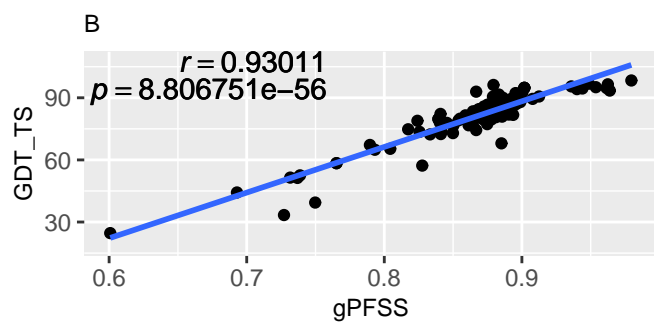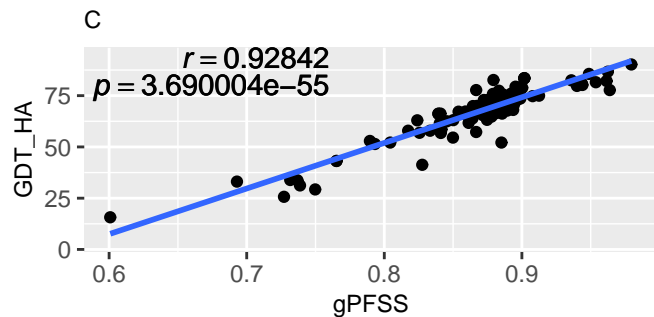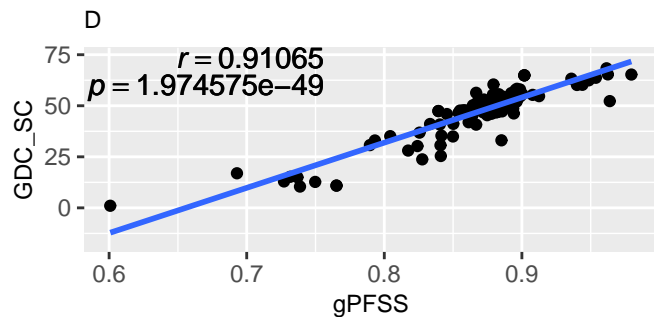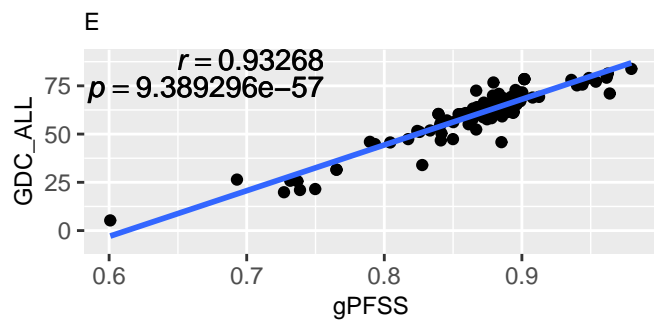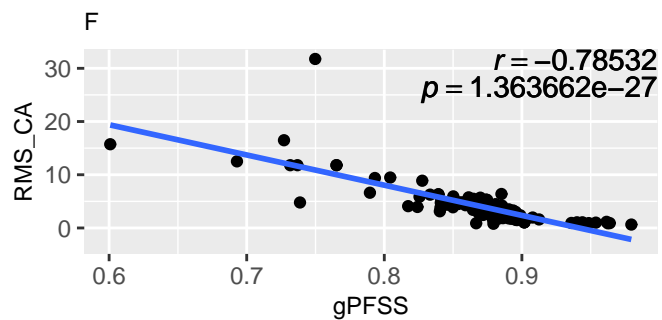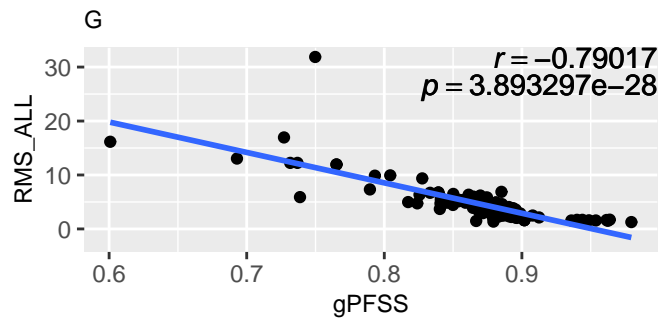

Supplement: Supplementary file 1 [file bioengineering-10-01377-s001.zip › Figure_S3_Plot_of_Metrics_gPFSS_TBMH.pdf]

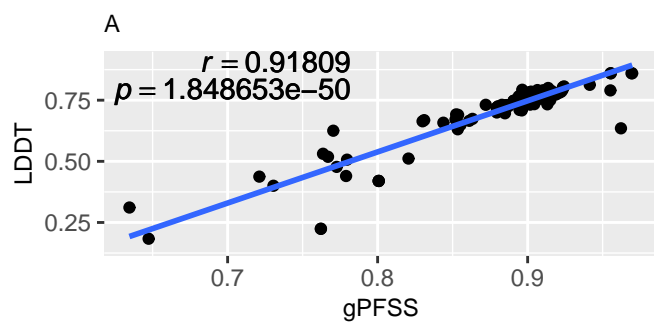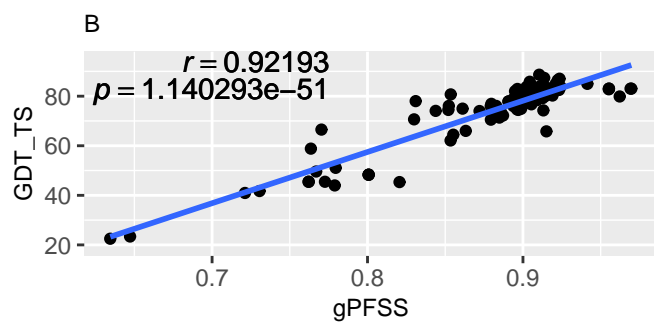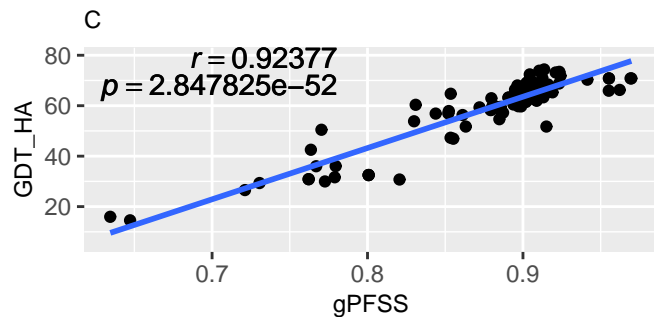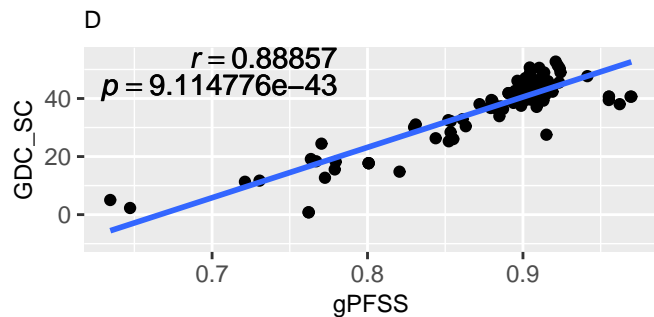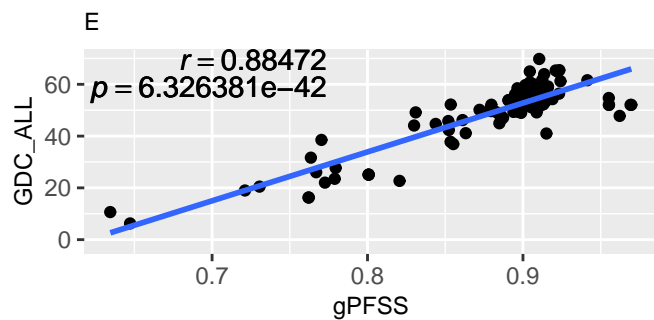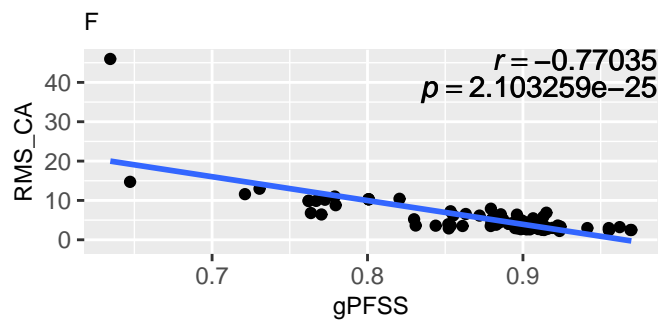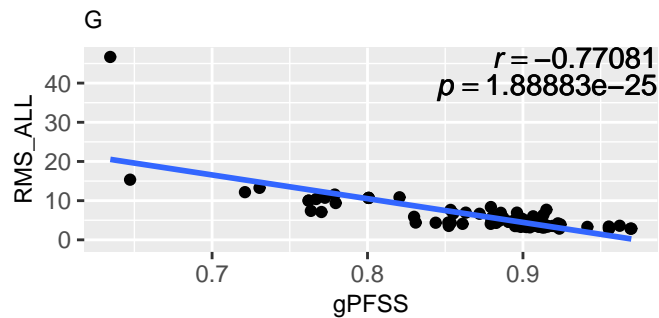

Supplement: Supplementary file 1 [file bioengineering-10-01377-s001.zip › Figure_S4_Plot_of_Metrics_gPFSS_FM_TBM.pdf]

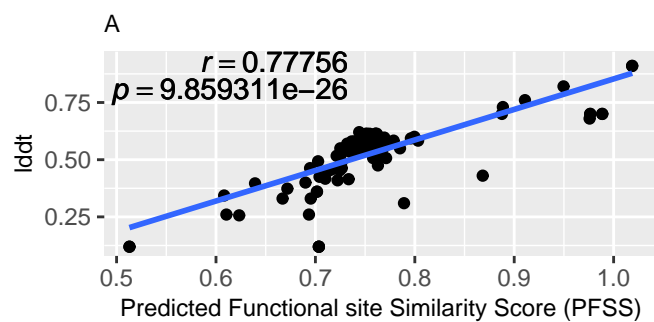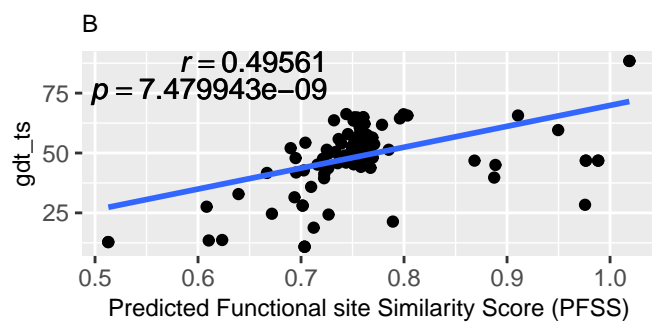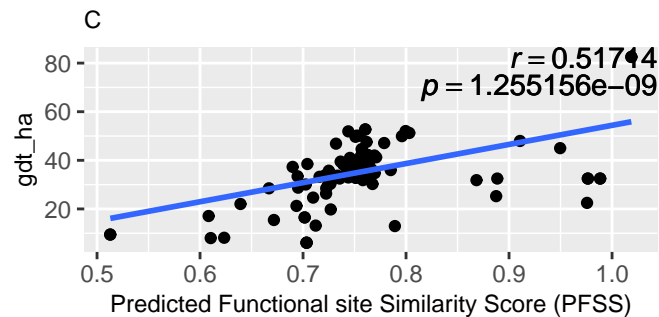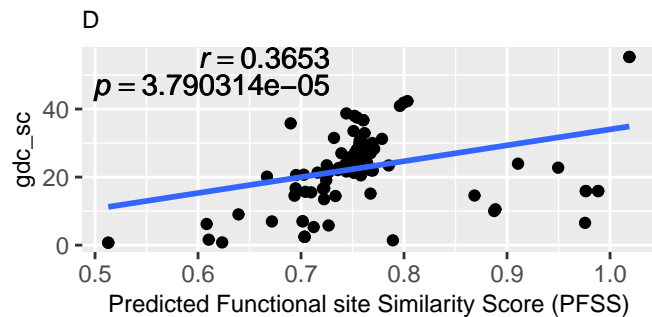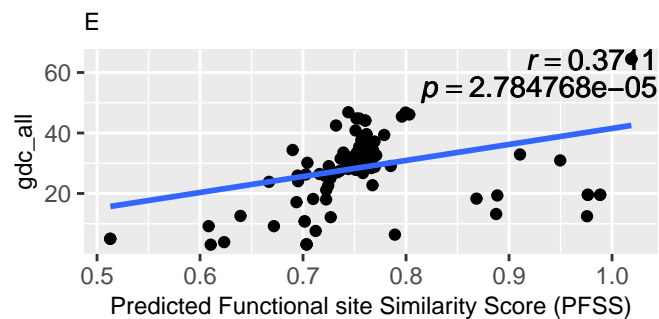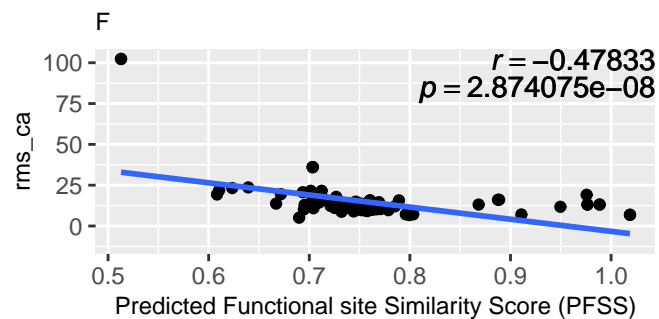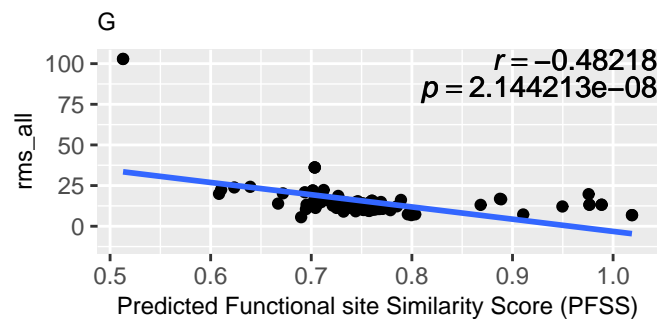

Supplement: Supplementary file 1 [file bioengineering-10-01377-s001.zip › Figure_S5_Plot_of_Metrics_gPFSS_Other.pdf]
